# Two-decade clinicopathological spectrum of gliomas at a tertiary center in LMIC: histological profile and the molecular diagnostic gap

**DOI:** 10.3389/fonc.2026.1909105

**Published:** 2026-09-15

**Authors:** Suleiman S. Daoud, Atef F. Hulliel, Sultan Jarrar, Amer Jaradat, Lina Ayman Alababneh, Adam Abdallah

**Affiliations:** 1 Neurosurgery Department, Faculty of Medicine, Jordan University of Science & Technology, Irbid, Jordan; 2 Department of Neurosurgery, Brain and Spine, University of South Florida Morsani College of Medicine, Tampa, FL, United States

**Keywords:** glioblastoma, glioma, IDH, low- and middle-income countries, molecular diagnostics, WHO classification

## Abstract

**Introduction:**

Diffuse gliomas are the most common malignant primary central nervous system (CNS) tumors, and contemporary World Health Organization (WHO) criteria require molecular markers, mainly isocitrate dehydrogenase (IDH) status and 1p/19q codeletion, for an integrated diagnosis. Access to these tests remains uneven in low- and middle-income settings. Data describing the glioma spectrum and the practical impact of limited molecular testing in Jordan are scarce.

**Method:**

We retrospectively retrieved all glioma-related surgical pathology specimens accessioned between 2003 and 2024 at a tertiary academic center in Jordan through a structured text search. Histological category, WHO grade, anatomic site, immunohistochemical (IHC) profile, Ki-67 index, and documented IDH/1p-19q status were extracted from the free-text reports. After exclusion of non-neoplastic, non-glial, and unclassifiable records, 430 specimens from 332 patients formed the analytic cohort. Ki-67 across grades was compared with the Kruskal-Wallis test and categorical age associations with the chi-square test.

**Results:**

The median age was 48 years (IQR 28-63; range 3-98) with a male predominance (male-to-female ratio 1.30). Glioblastoma was the most frequent diagnosis (174/430; 40.5%), followed by non-pilocytic astrocytoma (17.9%) and unspecified diffuse glioma (12.8%). The Ki-67 index increased significantly across WHO grades (median 3%, 2%, 20%, and 30% for grades I-IV; Kruskal-Wallis p < 0.0001). An actual IDH result was documented for only 10 specimens (2.3% overall; 3.0% of 329 diffuse gliomas), while reports explicitly noted that testing was indicated but unavailable in a further 17.4%. Glioblastoma was almost exclusive to adults and pilocytic astrocytoma to children (both p < 0.0001). Annual case volume rose from approximately 15 to 25 specimens per year, yet documented IDH results appeared only after 2017 and in fewer than 5% of recent specimens.

**Conclusion:**

Over two decades, gliomas at our center were diagnosed predominantly on morphology and a limited IHC panel. The near-absence of molecular results means the large majority of diffuse gliomas cannot be fully classified by current WHO standards, a concrete diagnostic-capacity gap with direct implications for prognostication, treatment stratification, and trial eligibility. The findings support investment in accessible molecular neuropathology, including surrogate IHC and regional referral testing.

## 1 Introduction

Tumors of the central nervous system (CNS) are associated with substantial morbidity and mortality, and gliomas represent a major category of primary malignant CNS neoplasms (1). Among them, glioblastoma is both the most common and the most lethal, with a median survival that remains under two years despite maximal multimodal therapy (2). Gliomas are a heterogeneous group of tumors, and accurate histological and molecular diagnosis is essential for prognostication and guiding treatment decisions (3).

The framework for that diagnosis has changed fundamentally over the past decade. The 2016 revision of the WHO Classification of Tumors of the CNS was the first to incorporate molecular parameters alongside histology, formally introducing the concept of an integrated diagnosis (4). The 2021 fifth-edition classification (WHO CNS5) extended this principle, making defined genetic alterations obligatory for the diagnosis and grading of most diffuse gliomas (5). In this scheme, the mutational status of isocitrate dehydrogenase (IDH) separates IDH-mutant astrocytomas from IDH-wildtype glioblastoma and carries strong prognostic weight (6), while whole-arm 1p/19q codeletion is required to designate an oligodendroglioma (7). O6-methylguanine-DNA methyltransferase (MGMT) promoter methylation further predicts benefit from alkylating chemotherapy (8). Consequently, a morphological diagnosis alone is now considered incomplete for diffuse gliomas, with direct consequences for clinical-trial eligibility and access to targeted agents (9).

These advances rely on access to specialized diagnostic resources, including immunohistochemistry, molecular sequencing, and copy-number analysis. However, access to these technologies remains uneven, and significant gaps in neuro-oncology diagnostic and treatment capacity continue to exist between high-income countries and resource-limited regions (10), at the same time, an increasing share of the global burden of CNS tumors falls on regions where access to advanced molecular testing remains limited (11).

Where molecular assays are unavailable, surrogate immunohistochemical markers such as IDH1 R132H and ATRX can partially bridge the gap, but they cannot fully replicate sequencing-based classification (12). The practical result is that many tumors in these settings receive a histological label that current criteria would consider provisional.

Epidemiological and pathological data on gliomas from the Middle East region remain comparatively limited, and most published series originate from centers with full molecular capability. Pediatric gliomas, which are biologically and clinically distinct from their adult counterparts, are similarly underrepresented in regional datasets (13).

To address this gap, we reviewed a 20-year institutional cohort of CNS gliomas from a tertiary academic center. We aimed, first, to characterize the clinicopathological spectrum of these tumors, including their histological types, WHO grades, anatomical distribution, immunophenotypic features, and age profile. Second, we sought to assess how many diffuse gliomas could be fully classified according to current WHO criteria, taking into account the extent of molecular testing available in our setting over the study period.

## 2 Methods and materials

### 2.1 Study design and setting

This was a retrospective, observational study conducted at a tertiary academic referral hospital in Jordan. The study was approved by the institutional review board. given the retrospective use of de-identified archival pathology data, the requirement for individual informed consent was waived.

### 2.2 Case identification

All surgical pathology reports accessioned between January 2003 and December 2024 were searched within the laboratory information system using a structured free-text query. The query returned 492 specimens.

### 2.3 Data extraction

For each specimen, the following variables were extracted from the report text: age, sex, anatomic site and laterality, specimen type (biopsy, resection, or intraoperative frozen section), final histological diagnosis, stated WHO grade, immunohistochemical stains referenced (including GFAP, p53, ATRX, OLIG2, and synaptophysin), numeric Ki-67 proliferation index where reported, and any documented IDH or 1p/19q result. Histological category was assigned from the final diagnosis line. WHO grade was recorded as stated; for glioblastoma and pilocytic astrocytoma, grade was assigned by definition (IV and I, respectively). IDH and 1p/19q findings were classified into three mutually exclusive states: an actual reported result; a report explicitly recommending the test or noting it was unavailable; or no mention.

Each specimen was assigned to a single histological category using the following operational rules: (i) any report stating glioblastoma or GBM was classed as glioblastoma; (ii) tumors designated pilocytic astrocytoma were separated from all other (non-pilocytic) astrocytomas; (iii) tumors labelled oligodendroglioma, ependymoma, or glioneuronal tumor/ganglioglioma were assigned to the corresponding named category; (iv) a diffuse glial tumor lacking a committed subtype was classed as diffuse glioma, unspecified subtype; and (v) embryonal (medulloblastoma) specimens were retained as a distinct, explicitly labelled category. Two reviewers independently re-examined each case and re-assigned it to the corresponding 2021 WHO CNS5 category on the basis of the available histological and immunohistochemical information, recording separately whether the molecular data required for a fully integrated diagnosis were present, recommended-but-unavailable, or not addressed; discrepancies were resolved by consensus.

### 2.4 Cohort definition

Of the 492 retrieved specimens, 62 were excluded as non-neoplastic (e.g., reactive gliosis, negative cytology), non-glial (e.g., schwannoma, metastatic or other tumors captured by the keyword search), or unclassifiable. The final analytic cohort comprised 430 specimens from 332 unique patients.

The keyword strategy was intentionally broad to capture the full range of CNS tumors accessioned under glial-associated terminology; consequently, four medulloblastoma specimens (0.9%) were returned. Although medulloblastoma is an embryonal tumor rather than a glioma, these cases were retained as a small, explicitly labelled category to preserve transparency of the search output. Because they are non-glial and none carried a molecular result, they are excluded from the diffuse-glioma denominator used for the molecular-gap analysis (n = 329).

### 2.3 Statistical analysis

Continuous variables are summarized as mean ± standard deviation and median with interquartile range (IQR); categorical variables as counts and percentages. To avoid over-counting patients who contributed more than one specimen, demographic characteristics are reported at the patient level (n = 332) and pathological characteristics at the specimen level (n = 430), as indicated. Because some patients contributed more than one specimen and no clinical outcomes were available, all pathological and molecular proportions represent specimen-level distributions. The Ki-67 index across WHO grades was compared using the Kruskal-Wallis test. Associations between age category (pediatric < 18 years versus adult) and tumor type were tested with the chi-square test. For pediatric-versus-adult comparisons, effect sizes were expressed as odds ratios (ORs) with 95% CIs and Cramér’s V, and Fisher’s exact test was applied where expected cell counts were small. Temporal trends in the proportion of specimens with a documented IDH result across the three eras were tested with the Cochran–Armitage test for trend, and the trend in annual specimen volume was modelled by Poisson regression (incidence-rate ratio (IRR) per calendar year), with the Spearman rank correlation as a sensitivity check. Binomial proportions are reported with 95% CIs. A two-sided p value < 0.05 was considered statistically significant. All analyses were performed using IBM SPSS Statistics for Windows, version 26.0.

## 3 Results

### 3.1 Patient characteristics

The analytic cohort comprised 430 specimens from 332 patients accessioned over the 21-year period (**Table 1**). The median age was 48 years (IQR 28-63), with a mean of 46.7 ± 21.7 years and a range of 3 to 98 years. Among patients with recorded sex (n = 322), 182 (56.5%) were male and 140 (43.5%) female, giving a male-to-female ratio of 1.30. Adults aged 40–64 years formed the largest age stratum (132 patients, 41.0%), followed by young adults aged 18–39 years (79, 24.5%) and older adults aged ≥ 65 years (75, 23.3%); 36 patients (11.2%) were pediatric (< 18 years).

**Table 1:** Demographic and clinical characteristics of the cohort.

Characteristic	Value	% of known
Patients, n	332	
Total specimens, n	430	
Sex, Male	182	56.5%
Sex, Female	140	43.5%
Sex, not recorded	10	
Male: Female ratio	1.30	
Age, mean ± SD (years)	46.7 ± 21.7	
Age, median [IQR] (years)	48 (X28–63)	
Age range (years)	3–98	
Pediatric (<18 y)	36	11.2%
Young adult (18–39 y)	79	24.5%
Adult (40–64 y)	132	41.0%
Older adult (≥65 y)	75	23.3%

Demographics reported at patient level (n = 332). Percentages for sex and age groups are of patients with the variable recorded (sex known n = 322; age known n = 322).

### 3.2 Histopathological spectrum

Glioblastoma was the single most common diagnosis, accounting for 174 of 430 specimens (40.5%) (**Table 2**). Non-pilocytic astrocytoma was next (77; 17.9%), followed by diffuse glioma of unspecified subtype (55; 12.8%), pilocytic astrocytoma (42; 9.8%), ependymoma (41; 9.5%), oligodendroglioma (23; 5.3%), glioneuronal tumor or ganglioglioma (14; 3.3%), and medulloblastoma (4; 0.9%) (**Figure 1**).

**Table 2:** Histopathological distribution of specimens.

Tumor category	n	%
Glioblastoma	174	40.5%
Astrocytoma, non-pilocytic	77	17.9%
Diffuse glioma, unspecified subtype	55	12.8%
Pilocytic astrocytoma	42	9.8%
Ependymoma	41	9.5%
Oligodendroglioma	23	5.3%
Glioneuronal tumor/ganglioglioma	14	3.3%
Medulloblastoma	4	0.9%
Total	430	100%

Specimen-level (n = 430). “unspecified subtype” denotes a diffuse glioma where the report did not commit to a definitive subtype.

**Figure 1:**
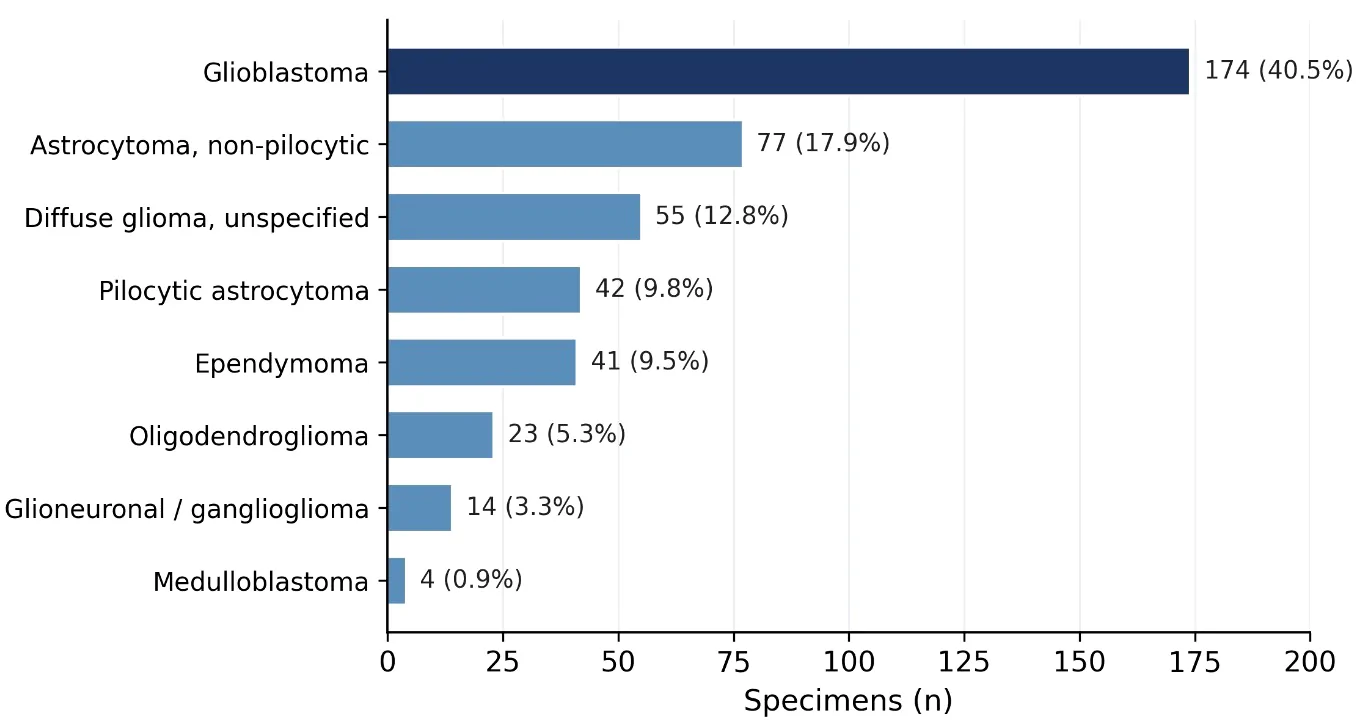
Horizontal bar chart comparing brain tumor specimen counts.

### 3.3 Tumor grade distribution

Across the cohort, WHO grade IV was the most frequent (178 specimens), followed by grade II (65), grade I (58), and grade III (46); grade was not stated in 83 specimens (19.3%) (**Table 3**). The grade distribution tracked histology as expected: pilocytic astrocytomas were almost entirely grade I (41/42), non-pilocytic astrocytomas were distributed across grades II and III (32 and 21, respectively), and glioblastomas were grade IV (173/174). Grade was most often unstated among unspecified diffuse gliomas (40/55) (**Figure 2**).

**Table 3:** WHO grade distribution by tumor category.

Tumor category	I	II	III	IV	Not stated
Glioblastoma	0	0	1	173	0
Astrocytoma, non-pilocytic	4	32	21	3	17
Pilocytic astrocytoma	41	1	0	0	0
Ependymoma	6	14	10	0	11
Oligodendroglioma	0	10	8	0	5
Diffuse glioma, unspecified	0	7	6	2	40
Glioneuronal/ganglioglioma	7	1	0	0	6
Medulloblastoma	0	0	0	0	4
All specimens	58	65	46	178	83

Specimen-level (n = 430). For glioblastoma and pilocytic astrocytoma, grade was assigned by definition (IV and I).

**Figure 2:**
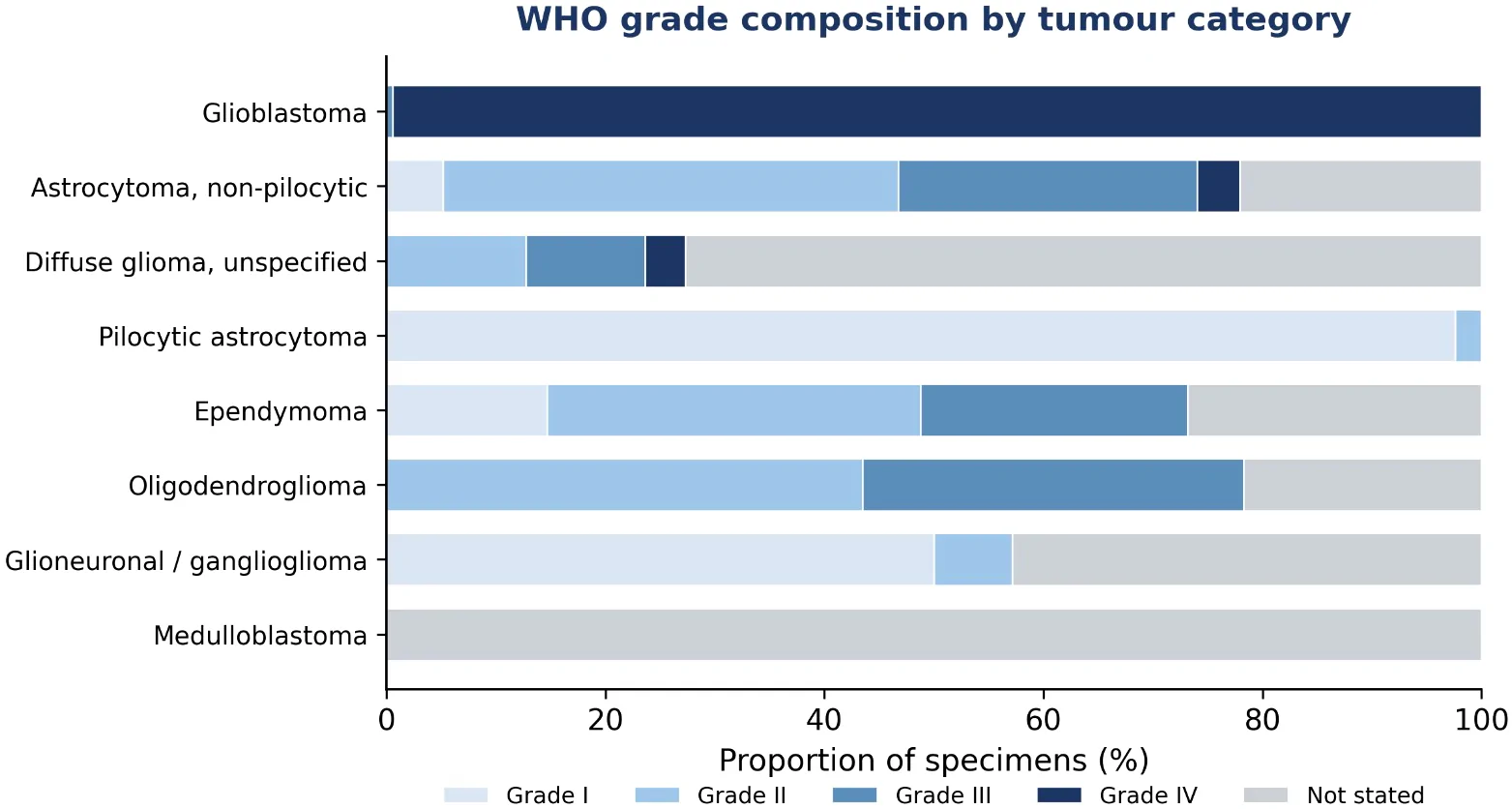
Horizontal bar chart illustrating the percentage composition of WHO grades.

### 3.4 Anatomic distribution

Among specimens with a documented site, supratentorial lobar locations predominated, temporal (70), parietal (60), frontal (46), and occipital (17) lobes, while infratentorial sites included the cerebellum (36), other posterior fossa locations (28), and brainstem (4) (**Table 4**). Spinal (23), ventricular or intraventricular (26), thalamic (11), optic-pathway (7), and sellar (6) locations were also recorded. Laterality, where stated, was left in 129 and right in 94 specimens. Anatomic site was not stated in 143 specimens (33.3%) (**Figure 3**).

**Table 4:** Anatomic distribution of tumors.

Site (specimens mentioning site)	n	Compartment
Temporal lobe	70	Supratentorial
Parietal lobe	60	Supratentorial
Frontal lobe	46	Supratentorial
Occipital lobe	17	Supratentorial
Cerebellum	36	Infratentorial
Posterior fossa (other)	28	Infratentorial
Brainstem	4	Infratentorial
Spinal/spinal cord	23	Spinal
Ventricular/intraventricular	26	Midline/other
Thalamus	11	Midline/other
Optic pathway	7	Midline/other
Sellar region	6	Midline/other
Site not stated	143	—

**Figure 3:**
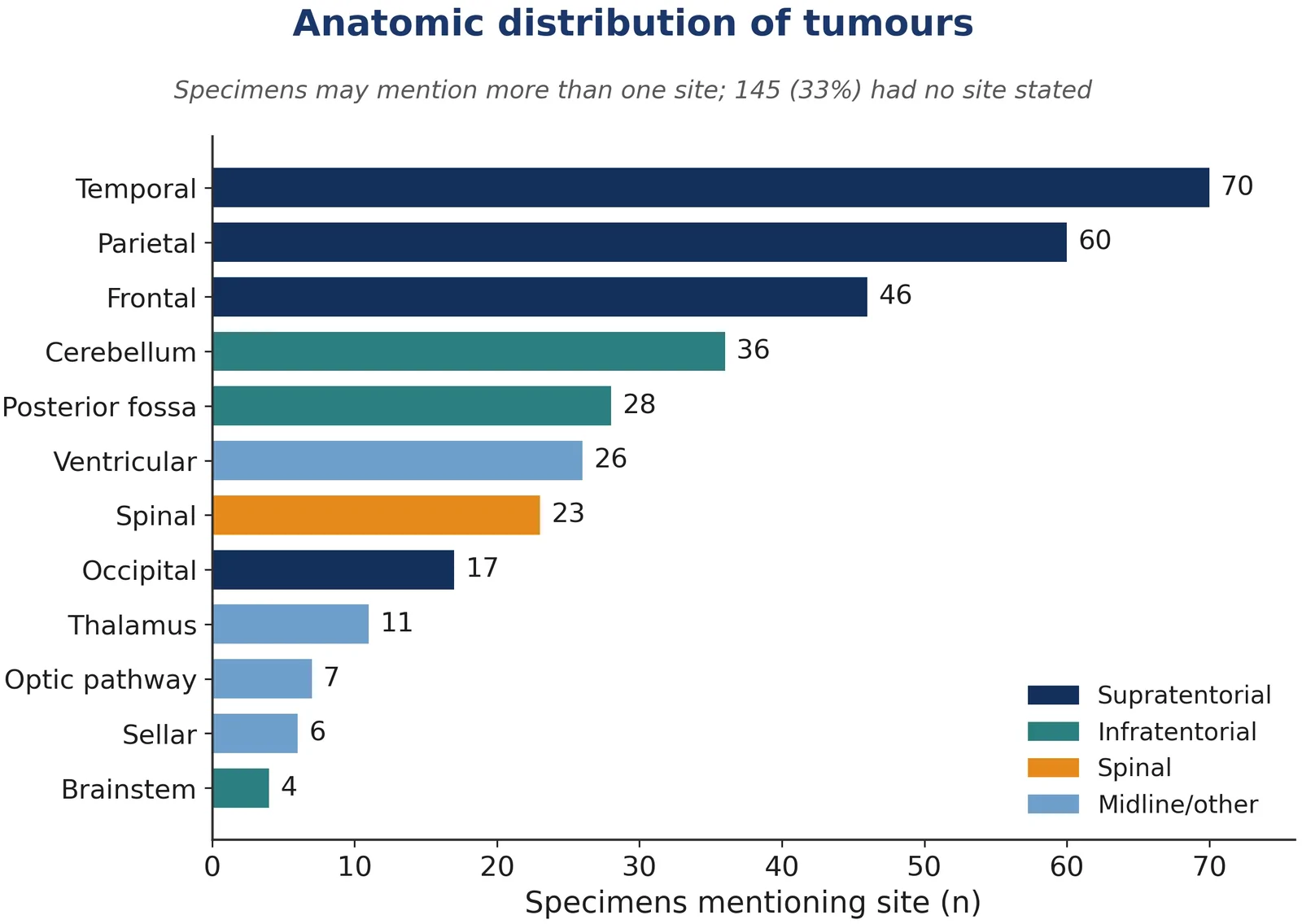
Horizontal bar chart illustrating the anatomic distribution of tumors by region.

### 3.5 Immunohistochemistry and proliferation index

GFAP was the most frequently referenced immunostain (220 specimens; 51%), followed by p53 (147; 34%), synaptophysin (46; 11%), ATRX (26; 6%), and OLIG2 (6; 1%) (**Table 5a**). A numeric Ki-67 proliferation index was reported for 74 specimens and increased markedly across WHO grades, with median values of 3% (grade I), 2% (grade II), 20% (grade III), and 30% (grade IV) (**Table 5b**; **Figure 4**). This gradient was statistically significant (H = 41.9; p < 0.0001).

**Table 5:** Immunohistochemistry use and Ki-67 proliferation index by grade.

5a. Immunohistochemical stains referenced						
Marker			Specimens, n		% of cohort	
GFAP			220		51%	
p53			147		34%	
Synaptophysin			46		11%	
ATRX			26		6%	
OLIG2			6		1%	

5b. Ki-67 index by WHO grade						
WHO grade	n with value		Median (%)		Range (%)	
I	8		3		1–5	
II	18		2		1–30	
III	7		20		5–80	
IV	41		30		5–90	

A numeric Ki-67 value was recorded in 74 specimens. Kruskal–Wallis test across grades: H = 41.9, p < 0.0001.

**Figure 4:**
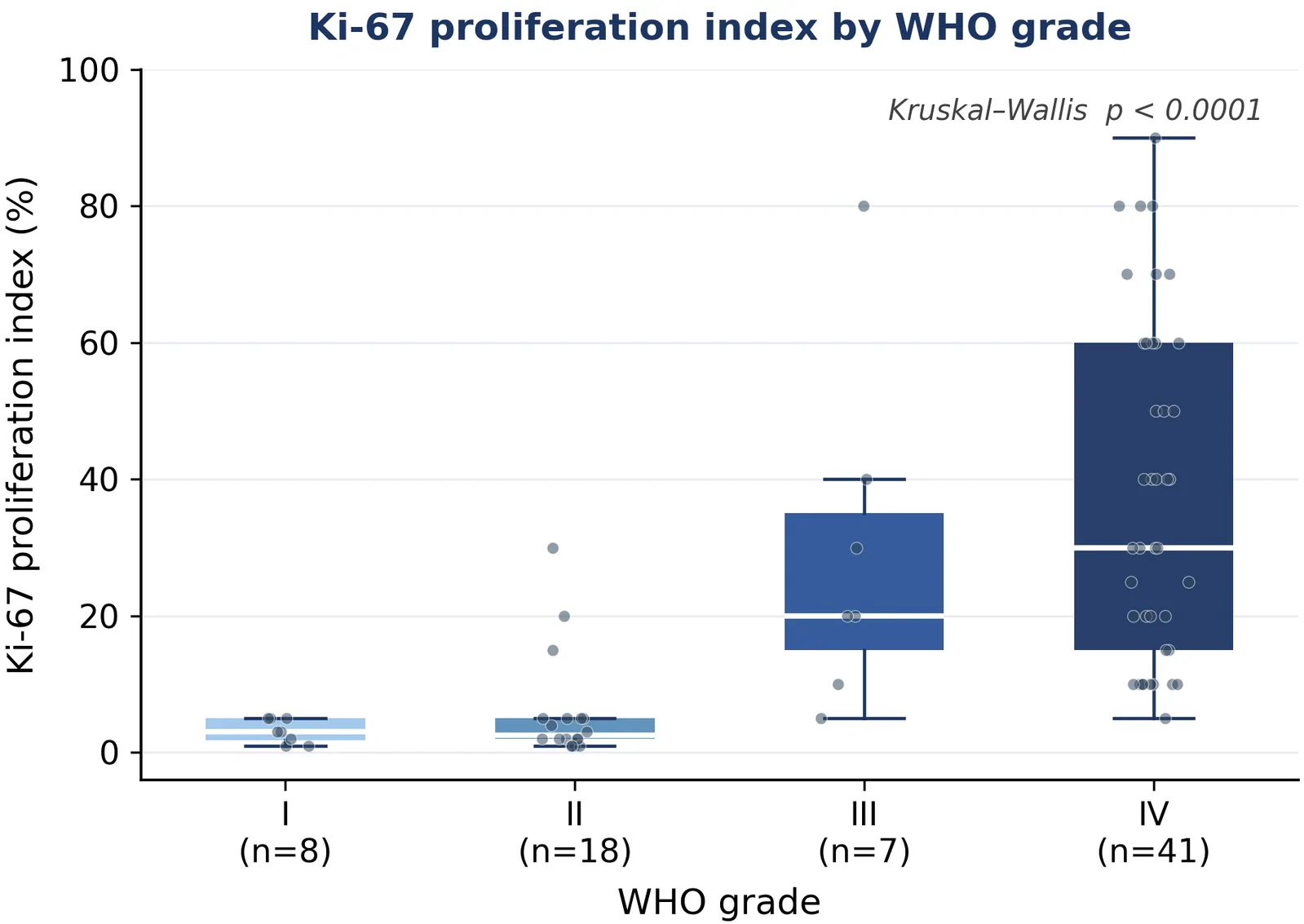
Box plot graphic compares Ki-67 proliferation index percentages across WHO grades.

### 3.6 Molecular diagnostic status

An actual IDH result was documented in only 10 specimens (2.3% of the cohort), comprising 6 IDH-mutant and 4 IDH-wildtype cases (**Table 6**; **Figure 5**). In a further 75 specimens (17.4%), the report explicitly stated that IDH testing was indicated or mandatory but unavailable; IDH was not addressed in the remaining 345 specimens (80.2%). A 1p/19q codeletion result was reported in 11 specimens (2.6%) and flagged as recommended but unavailable in 15 (3.5%). When restricted to diffuse gliomas requiring molecular characterization (n = 329), an IDH result was available for only 10 specimens (3.0%). Overall, at least one molecular test was explicitly documented as needed or unavailable in 77 specimens (17.9%).

**Table 6:** Molecular diagnostic gap: availability of integrated-diagnosis markers.

Marker/status	n	%
IDH, actual result reported	10	2.3%
IDH, recommended but unavailable	75	17.4%
IDH, not addressed	345	80.2%
1p/19q, actual result reported	11	2.6%
1p/19q, recommended but unavailable	15	3.5%
1p/19q, not addressed	404	94.0%
Any molecular test flagged needed/unavailable	77	17.9%
Diffuse gliomas with an IDH result (of n = 329)	10	3.0%

Specimen-level (n = 430). “Recommended but unavailable” denotes reports explicitly stating that IDH/1p-19q testing was indicated or mandatory but could not be performed in-house.

**Figure 5:**
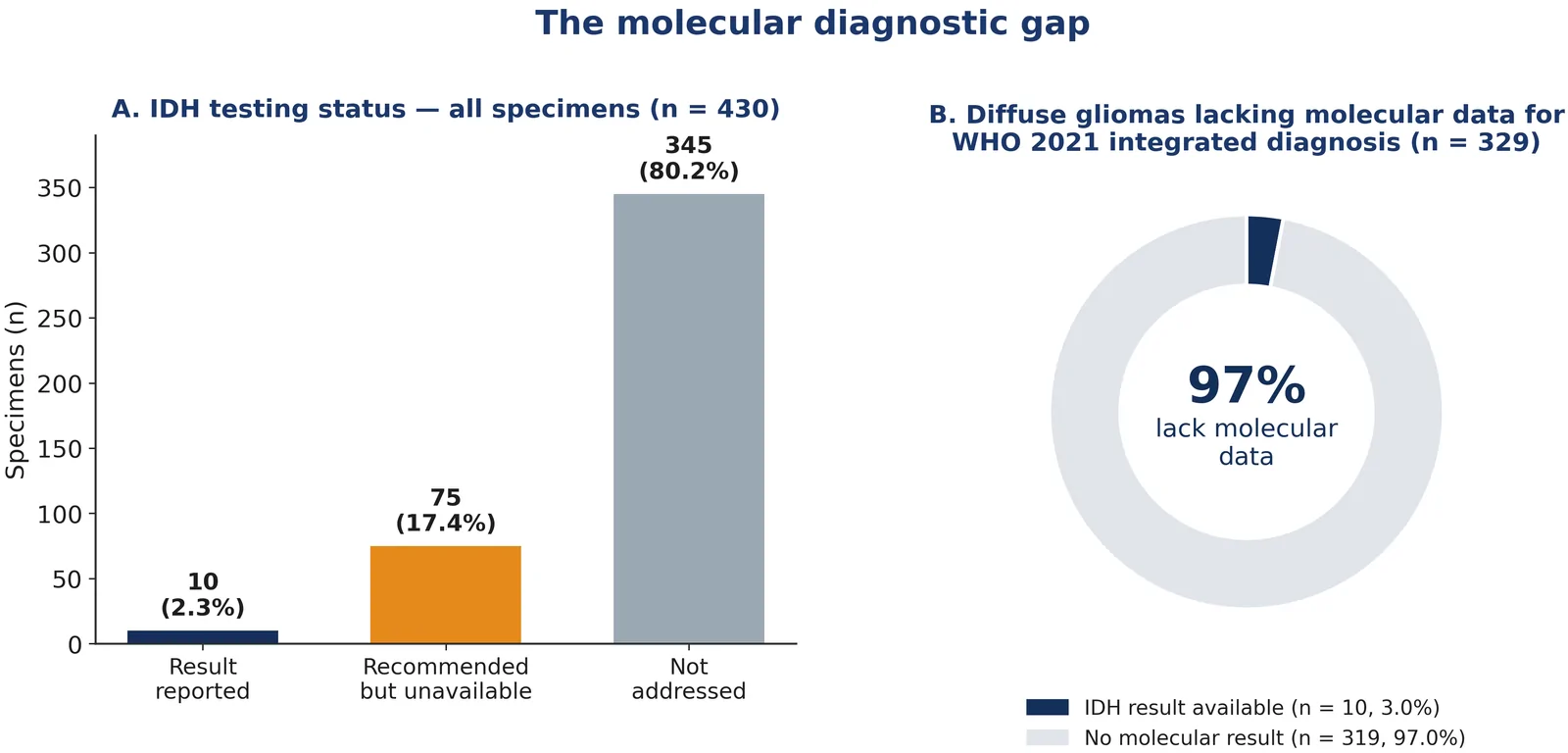
Bar chart and doughnut chart illustrating the molecular diagnostic gap in glioma specimens.

### 3.7 Temporal trends

Annual specimen volume rose across the study period, from a mean of 15.3 per year in 2003-2009 (107 specimens) to 17.6 per year in 2010-2016 (123 specimens) and 25.0 per year in 2017-2024 (200 specimens) (**Table 7**). Documented IDH results were absent in the earliest era (0/107), appeared in a single specimen in 2010-2016 (1/123; 0.8%), and reached 9 of 200 specimens (4.5%) in 2017-2024 (Wilson 95% CIs 0.0–3.5%, 0.1–4.5%, and 2.4–8.3%, respectively). This increase in documented IDH testing was statistically significant on the Cochran–Armitage test for trend (z = 2.68; p = 0.007). Annual specimen volume also rose significantly across the 21-year period (Poisson IRR 1.031 per calendar year, 95% CI 1.016–1.047; p < 0.001; Spearman ρ = 0.52, p = 0.013). (**Figure 6**).

**Table 7:** Temporal trends across three eras.

Era	Specimens, n	Mean/yr	IDH result, n (%)
2003–2009	107	15.3	0 (0.0%)
2010–2016	123	17.6	1 (0.8%)
2017–2024	200	25.0	9 (4.5%)

Specimen-level. “IDH result” = specimens with an actual reported IDH result in that era.

**Figure 6:**
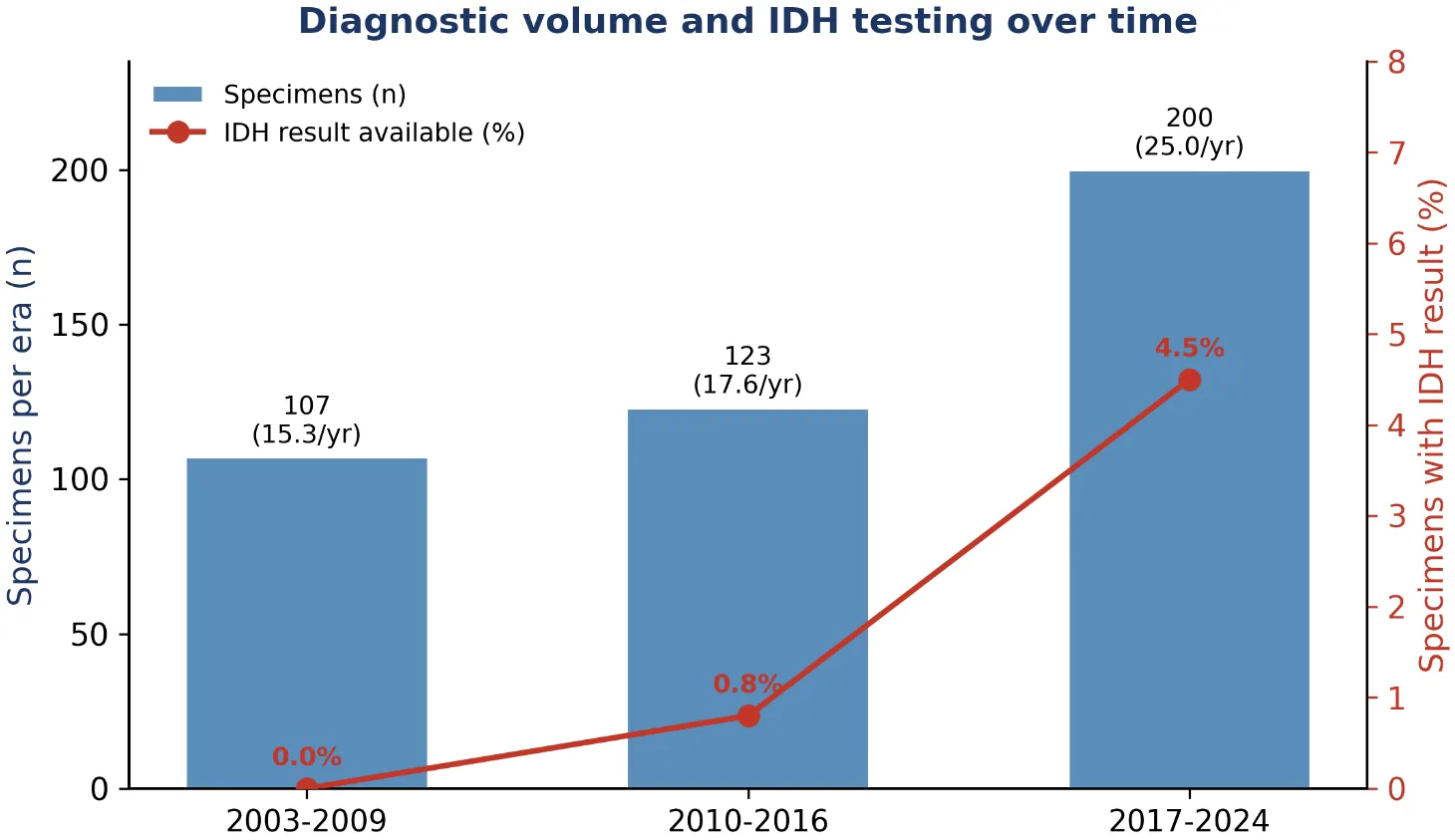
Bar and line graph showing diagnostic specimen volume and IDH testing results from 2003 to 2024.

### 3.8 Age-stratified distribution

Among specimens with known age (n = 420), tumor type differed markedly between children and adults (**Table 8**). In the pediatric group (n = 43), the most frequent diagnoses were pilocytic astrocytoma (30%), ependymoma (26%), and non-pilocytic astrocytoma (21%). In adults (n = 377), glioblastoma predominated (44%), followed by non-pilocytic astrocytoma (18%) and unspecified diffuse glioma (13%). Glioblastoma was almost exclusively an adult tumor (165 adults versus 2 pediatric specimens; (χ² = 24.7, df = 1, p < 0.0001; Fisher’s exact p < 0.0001; OR for the pediatric group 0.06, 95% CI 0.02–0.26; Cramér’s V = 0.24), whereas pilocytic astrocytoma was significantly increased in children, 13 of 43 pediatric specimens (30.2%) versus 29 of 377 adult specimens (7.7%), (χ² = 21.8, df = 1, p < 0.0001; Fisher’s exact p < 0.0001; OR 5.20, 95% CI 2.45–11.04; Cramér’s V = 0.23). (**Figure 7**).

**Table 8:** Pediatric versus adult tumor distribution.

Tumor category	Pediatric (<18 y)	Adult (≥18 y)
Pilocytic astrocytoma	13	29
Ependymoma	11	30
Astrocytoma, non-pilocytic	9	66
Glioneuronal/ganglioglioma	3	11
Diffuse glioma, unspecified	5	49
Glioblastoma	2	165
Oligodendroglioma	0	23
Medulloblastoma	0	4
Total specimens	43	377

Specimen-level, age known (n = 420). Pediatric vs adult association: glioblastoma χ² p < 0.0001; pilocytic astrocytoma χ² p < 0.0001.

**Figure 7:**
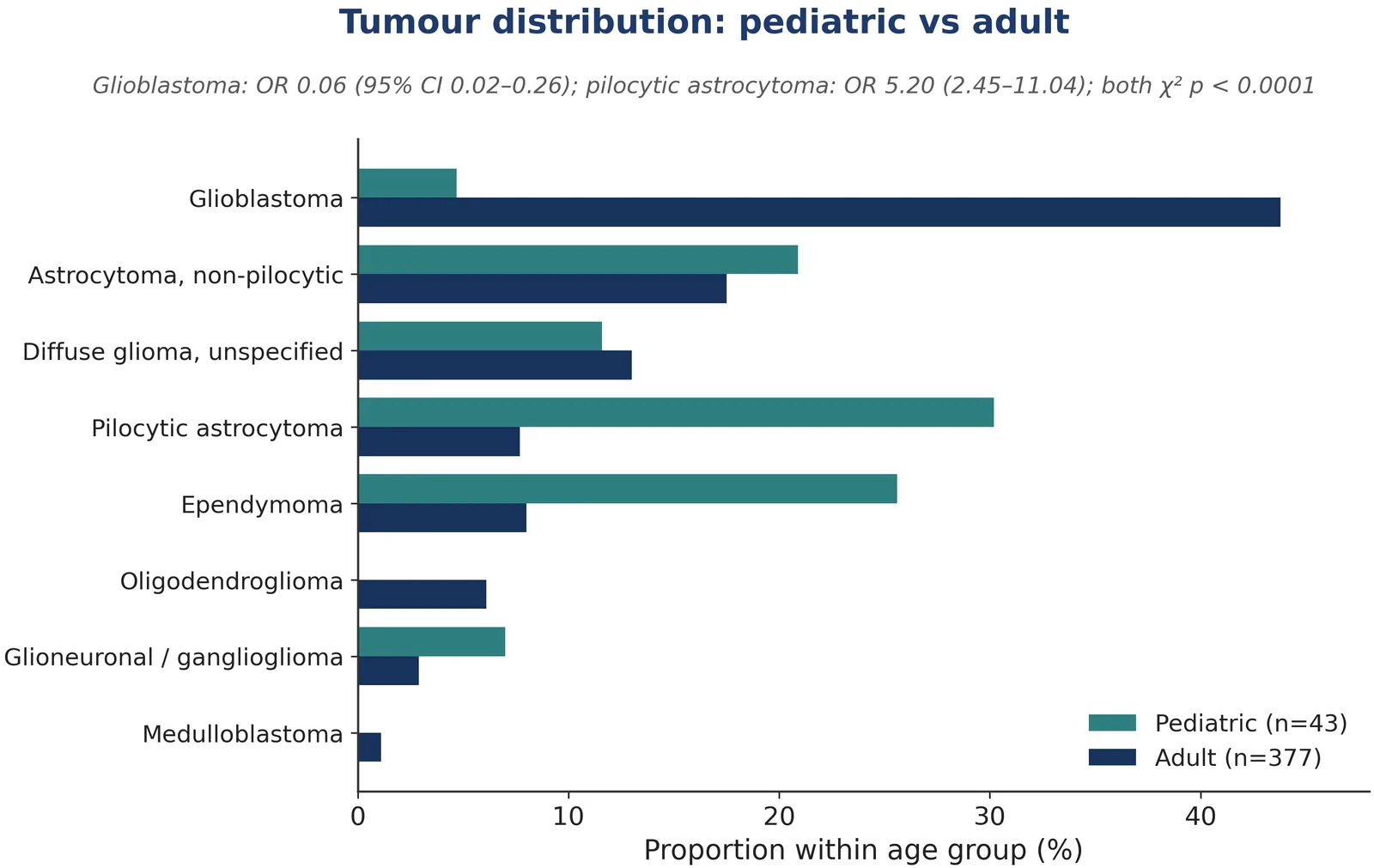
Horizontal bar chart comparing the proportion of different brain tumour types in pediatric and adult age groups.

## 4 Discussion

The predominance of glioblastoma, accounting for 40.5% of specimens, is consistent with large population-based registries in which glioblastoma represents roughly half of malignant gliomas and the most common malignant primary brain tumor (14). The observed male predominance (overall male-to-female ratio 1.30, rising to 1.74 among glioblastomas) accords with the consistent male excess described for glioblastoma worldwide (14). Our median age of 48 years across all gliomas is younger than the typical peak for glioblastoma alone, reflecting the inclusion of lower-grade and pediatric tumors in the denominator; age-specific analyses confirmed the expected concentration of glioblastoma in older adults (15). Regional series from neighboring countries have likewise reported glioma distributions broadly comparable to Western data, supporting the representativeness of our cohort (16, 17).

The significant stepwise increase in Ki-67 index across grades (medians of 3%, 2%, 20%, and 30% for grades I-IV) reproduces the proliferation-grade relationship repeatedly documented in glioma pathology and supports the prognostic relevance of Ki-67 as an adjunct to grading (18, 19). The slight overlap between grades I and II in our data is consistent with the known variability and limited discriminatory value of Ki-67 at the low-grade end, where its interpretation is most operator-dependent (20). These findings suggest that conventional histopathological evaluation, complemented by basic immunohistochemical markers, remains capable of generating diagnostically consistent results, although contemporary classification increasingly requires molecular testing for complete tumor characterization (21).

A key finding of our study is the persistent molecular diagnostic gap. With the WHO 2016 and 2021 classifications now placing molecular markers at the center of glioma diagnosis, determining IDH status is essential for distinguishing IDH-mutant astrocytomas from IDH-wildtype glioblastomas (5, 6), while the diagnosis of oligodendroglioma requires confirmation of 1p/19q codeletion (22). In our cohort, these determinations were essentially unavailable: only 2.3% of all specimens and 3.0% of diffuse gliomas had a documented IDH result, and reports frequently stated outright that the test was indicated but could not be performed locally. IDH-mutant gliomas carry a markedly better prognosis than their wildtype counterparts (23), and the distinction now also determines eligibility for IDH-targeted therapy, following trials demonstrating that the mutant-IDH inhibitor vorasidenib prolongs progression-free survival in residual or recurrent grade 2 IDH-mutant glioma (24). Likewise, MGMT promoter methylation predicts benefit from temozolomide, particularly in elderly patients, and informs treatment selection (24). Without these data, patients may be misclassified, could limit access to targeted agents, or excluded from clinical trials that mandate molecular eligibility.

Contemporary evidence confirm that IDH-wildtype glioblastoma continues to carry a substantially worse prognosis than IDH-mutant astrocytoma, and that molecularly defined glioblastoma can behave differently from its purely histological counterpart (25, 26). Because treatment data were not available in our cohort, we cannot directly quantify any downstream effect on therapy access; nevertheless, the absence of molecular results means that affected patients cannot be assigned an integrated WHO diagnosis and may not be identifiable for molecularly-defined clinical trials.

Our findings align with an expanding body of evidence showing that access to molecular neuro-oncology remains limited in many low- and middle-income countries. Financial constraints, limited laboratory infrastructure, and shortages of specialized personnel continue to hinder the routine implementation of molecular testing techniques, including sequencing and copy-number analysis (24). Several studies have suggested practical, stepwise diagnostic strategies for settings where full molecular profiling is not readily available. These approaches rely on a limited panel of surrogate immunohistochemical markers, such as IDH1 R132H, ATRX loss, and BRAF V600E, combined with selective referral of cases for external molecular testing. Together, these tiered workflows aim to approximate the integrated diagnostic framework recommended by the WHO classification while acknowledging real-world resource constraints in many laboratories (21, 27). The very low documented use of ATRX (6%) and OLIG2 (1%) in our series suggests that even these affordable surrogate markers were underutilized, indicating an opportunity for low-cost improvement before more expensive molecular platforms are considered. Establishing IDH1 R132H immunohistochemistry as a routine reflex stain for diffuse gliomas would, by itself, resolve a substantial fraction of the classification uncertainty that was observed. Several practical factors underlie the near-absence of in-house molecular testing at our center. Sequencing platforms and 1p/19q copy-number assays carry high capital and per-test reagent costs, while the relatively low local test volume makes in-house assays difficult to justify economically; procurement and supply-chain constraints for reagents and antibodies, the absence of dedicated molecular-pathology personnel, and the lack of a defined reimbursement pathway compound the problem.

A closer look at the timeline adds an important dimension. Even though the number of cases increased by about two-thirds over the study period, and IDH testing only started appearing after 2017, it was still documented in fewer than 5% of the most recent samples. This suggests that while awareness of molecular requirements is improving, access to the actual testing infrastructure has not kept pace. The pattern mirrors what has been reported in other regional settings, where advances in guidelines tend to move faster than the ability of laboratories to implement them in routine practice. The implication is that guideline adoption alone, absent investment in testing infrastructure or referral pathways, does not close the gap.

The age-stratified distribution further reinforces the fundamental biological divergence between pediatric and adult gliomas. The marked predominance of pilocytic astrocytoma in the pediatric cohort, contrasted with the strong scale of glioblastoma in adults, is consistent with established population-based epidemiology (28). In line with the WHO CNS5 classification, pediatric-type diffuse gliomas are now recognized as biologically discrete entities characterized by recurrent, pathway-specific molecular alterations, including MAPK pathway activation (e.g., BRAF variants), FGFR1-associated changes, and histone H3 mutations, underscoring their fundamental molecular separation from adult-type diffuse gliomas (29–31). This distinction has direct management implications, as pediatric low-grade gliomas increasingly benefit from targeted MAPK-pathway inhibition (32, 33).

Our findings highlight the practical need to expand molecular neuropathology services in similar resource-constrained settings. As an initial and relatively cost-effective measure, incorporating routine surrogate immunohistochemical markers, such as IDH1 R132H, ATRX, BRAF V600E, and selected markers for ependymoma, could substantially improve diagnostic precision. Over the longer term, establishing regional referral networks or centralized molecular diagnostic facilities may offer a feasible strategy for providing access to sequencing technologies and 1p/19q codeletion testing, allowing centers to benefit from advanced molecular classification without the substantial infrastructure requirements of developing comprehensive in-house testing capabilities. Prospective registries that capture molecular status and outcomes would allow the field to move beyond descriptive series toward survival and predictive analyses, which our dataset could not support. Future work should also evaluate the diagnostic and economic impact of introducing reflex surrogate testing and quantify how reclassification under WHO CNS5 alters management in resource-limited cohorts.

The main strengths of this study lie in its long, uninterrupted 21-year follow-up period and its relatively large, single-center cohort, which together offer a consistent overview of glioma diagnostics within a regional referral context. By systematically reviewing and reclassifying all available cases according to contemporary WHO criteria on the basis of the available histological and immunohistochemical data, we were able not only to describe but also to quantify the proportion of diffuse gliomas that could not be fully classified due to the lack of molecular testing, a metric that is rarely reported in similar settings. Moreover, the strong alignment of our findings with established epidemiological patterns, as well as the expected relationship between Ki-67 proliferation index and tumor grade, supports the reliability and internal consistency of the dataset.

Several limitations warrant emphasis. First, the study is retrospective and confined to a single institution, which may limit generalizability and introduce referral bias. Second, because most diagnoses predated the WHO 2021 classification and molecular data were largely absent, retrospective reclassification was constrained, indeed, that constraint is itself a core finding rather than only a limitation. Finally, the keyword-based retrieval, although broad, may have missed tumors recorded under atypical terminology or, conversely, captured non-glial lesions that we then excluded.

## 5 Conclusion

Over two decades, gliomas at our center were diagnosed predominantly on morphology supported by a limited immunohistochemical panel, with a histological and age distribution consistent with international experience. However, the molecular markers now required for an integrated WHO diagnosis were almost entirely unavailable, leaving the large majority of diffuse gliomas unclassifiable by current standards. This measurable diagnostic gap carries consequences for diagnostic classification, prognostication, referral pathways, and eligibility for molecularly-defined clinical trials. Strengthening accessible molecular neuropathology, beginning with affordable surrogate immunohistochemistry and regional referral testing, should be a priority for improving glioma care in this and similar settings.

## Data Availability

The data analyzed in this study is subject to the following licenses/restrictions: The datasets generated and/or analyzed during the current study are not publicly available due to institutional privacy and data protection regulations but are available from the corresponding author on reasonable request and with permission from the institutional review board. Requests to access these datasets should be directed to Suleiman S. Daoud, ssdaoud@just.edu.jo.
